# Metagenomic Insights into the Effects of Seasonal Temperature Variation on the Activities of Activated Sludge

**DOI:** 10.3390/microorganisms7120713

**Published:** 2019-12-17

**Authors:** Chenbing Ai, Zhang Yan, Han Zhou, Shanshan Hou, Liyuan Chai, Guanzhou Qiu, Weimin Zeng

**Affiliations:** 1 School of Metallurgy and Environment, Central South University, Changsha 410083, China; chenbingai@csu.edu.cn (C.A.);; 2 School of Minerals Processing and Bioengineering, Central South University, Changsha 410083, China; 3 Chinese National Engineering Research Center for Control and Treatment of Heavy Metal Pollution, Central South University, Changsha 410083, China; 4 Key Laboratory of Biometallurgy of Ministry of Education, Central South University, Changsha 410083, China; 5 College of Environmental Science and Engineering, Fujian Key Laboratory of Pollution Control & Resource Reuse, Fujian Normal University, Fuzhou 350007, China

**Keywords:** activated sludge, seasonal temperature variation, wastewater treatment, high-throughput sequencing, metagenome, microbial community

## Abstract

It is well acknowledged that the activities of activated sludge (AS) are influenced by seasonal temperature variation. However, the underlying mechanisms remain largely unknown. Here, the activities of activated sludge under three simulated temperature variation trends were compared in lab-scale. The TN, HN_3_-H, and COD removal activities of activated sludge were improved as temperature elevated from 20 °C to 35 °C. While, the TN, HN_3_-H, COD and total phosphorus removal activities of activated sludge were inhibited as temperature declined from 20 °C to 5 °C. Both the extracellular polymer substances (EPS) composition (e.g., total amount, PS, PN and DNA) and sludge index of activated sludge were altered by simulated seasonal temperature variation. The variation of microbial community structures and the functional potentials of activated sludge were further explored by metagenomics. *Proteobacteria*, *Actinobacteria*, *Acidobacteria* and *Bacteroidetes* were the dominant phyla for each activated sludge sample under different temperatures. However, the predominant genera of activated sludge were significantly modulated by simulated temperature variation. The functional genes encoding enzymes for nitrogen metabolism in microorganisms were analyzed. The enzyme genes related to ammonification had the highest abundance despite the changing temperature, especially for gene encoding glutamine synthetase. With the temperature raising from 20 °C to 35 °C. The abundance of *amoCAB* genes encoding ammonia monooxygenase (EC:1.14.99.39) increased by 305.8%. Meanwhile, all the enzyme genes associate with denitrification were reduced. As the temperature declined from 20 °C to 5 °C, the abundance of enzyme genes related to nitrogen metabolism were raised except for carbamate kinase (EC:2.7.2.2), glutamate dehydrogenase (EC:1.4.1.3), glutamine synthetase (EC:6.3.1.2). Metagenomic data indicate that succession of the dominant genera in microbial community structure is, to some extent, beneficial to maintain the functional stability of activated sludge under the temperature variation within a certain temperature range. This study provides novel insights into the effects of seasonal temperature variation on the activities of activated sludge.

## 1. Introduction

Activated sludge system is a traditional biological treatment technology that has been applied in more than 80% urban sewage treatment plants in the world ascribing to its appealing advantages of high processing capacity and excellent effluent quality [1]. Activated sludge (AS) is the core component of the activated sludge system. Activated sludge is a complex of consortium formed by various microorganisms together with their adsorbed organic and inorganic substances, which is the mainstay that mineralize and decompose organic pollutants in sewage treatment plants. The functional stability of activated sludge depends largely on the activity, composition and diversity of the microorganisms in activated sludge, which is usually influenced by various operating parameters and environmental factors, such as seasonal temperature variation [2,3]. Therefore, an in-depth study of microbial community structure of activated sludge under different operating parameters and environmental factors will provide insights for stabilizing and improving the efficiency of wastewater treatment. 

Operating parameters (e.g., BOD-SS load, volumetric load, substance (matrix) concentration, dissolved oxygen concentration) can be artificially modulated through certain equipment to ensure their fluctuation within a relatively stable range. However, it is not feasible to artificially control the water temperature that mainly depends on geographical location, weather conditions and season changing. On the other hand, water temperature can greatly affect the activated sludge purification reaction. The processes of degradation of BOD substances and the removal of COD, total N, NH_3_-N and total P by activated sludge are mediated by microorganisms, which are extremely sensitive to temperature. Therefore, the activity of activated sludge can be adversely affected by variable temperature that above or below the optimal temperature. In addition, the water temperature directly affects the sludge expansion and water viscosity which are important factors for the separation of activated sludge and water in the sedimentation tank. 

The empirical approach and microscopic observation methods are still the main methods to judge the performance of activated sludge adopted by many Chinese municipal sewage treatment plants, which are not only primitive but also subjective. In contrast, in-depth study of the microbial composition and metabolic potentials of activated sludge with culture-independent high-throughput sequencing technology will undoubtedly help to further reveal the mechanism underlying th operating process, and thereby achieve the goal of improving treatment efficiency and reducing treatment cost. Recently, metagenomic approaches based on high-throughput sequencing have been widely used to explore the structures and functions of microbial communities. This promising approach will provide a great opportunity and new insights to reveal composition of microbial communities, diversity of functional genes and novel genes in the environment [4]. Feng, J. et al. [5] studied the seasonal microbial variations of activated sludge from a full-scale wastewater treatment plant over four years using metagenomic approach and showed that beside temperature and salinity, other variables (such as SRT, DO, salinity, etc.) also play significant roles in shaping the overall activated sludge community structure. Guo, J. et al. [6] used metagenomic approach to unravel the community diversity and functional profiles within activated sludge from a full-scale SNPR WWTP and found that various key enzymes involved in N and P metabolisms could be annotated in the activated sludge. However, it was hard to distinguish the effects of each different variables on microbial community in a full-scale WWTP since a lot of uncontrollable or even undetectable influential factors were involved in such a pollutant-removing process. Thus, it is necessary to evaluate the adverse effects of temperature variation on the microbial community structure and the activities of activated sludge under controllable processing parameters. 

In this study, the activities of activated sludge were first evaluated in laboratory-scale reactors under controllable processing parameters besides the simulated temperature variation. Total genomic DNA samples were extracted from the activated sludge in reactors operated under different variable temperatures. The effect of temperature variation on the microbial community structure and functional genes of activated sludge was further explored by high-throughput sequencing and metagenomic analyzes. The intrinsic relationship between community structure and function of microbial under variable water temperature was proposed by metagenomics analysis. This study provides novel insights into the adverse effects of temperature variation on the microbial community structure and the activities of activated sludge.

## 2. Materials and Methods

### 2.1. Wastewater and Seed Sludge

The synthetic feeding medium used as simulated influent in reactor contained 400 mg/L glucose, 150 mg/L peptone, 46.4 mg/L NH_4_Cl, 2.3 mg/L KH_2_PO_4_, 22.5 mg/L MgSO_4_·H_2_O, 12 mg/L Na_2_HPO_4_·12H_2_O, 22.5 mg/L CaCl_2_ and 0.25 mg/L FeCl_3_·H_2_O. All the chemicals used in this study were analytic pure (Sinopharm Chemical Reagent Co., Ltd., shanghai, China). The seed mixed liquor suspended solids (MLSS) at aerobiotic phase was collected from the lab-scale sequencing batch reactor (SBR) (Supplementary Figure S1) that performed nutrient removal under a cycle time of 24 h with the artificially controlled temperature (20 ± 1 °C). Each cycle contained the following six sequential procedures: feed (instantaneous), anaerobic (2 h), aerobiotic (6 h), anoxia (2 h), effluent discharging (instantaneous, 80% water exchange, 2 L) after sediment (2 h), idle (12 h) process. The lab-scale SBR was operated for more than 2 months to achieve the steady-state as demonstrated by efficient and stable COD and TN removal efficiencies before the initiation of the simulated temperature variation processes.

### 2.2. The Lab-Scale SBR and the Operating Conditions

Three 3 L beakers were used as bioreactors for the operation of SBR activated sludge process with an effective working volume of 3 L. An aquarium air pump (Hailea ACO-9602, Guangzhou, China) was used to supply air through a porous stone diffuser that located at the bottom of reactor with an air flow rate of 1.5 L/min. Even distribution of wastewater was achieved through the paddles mixing at a speed of 100 rpm. Reactors were covered with aluminum foil to prevent evaporation [7]. 

The activated sludge was added to SBRs at a final concentration of approximately 2500 mg/L in the synthetic wastewater and operated under the conditions as mentioned above. The sludge concentrations in all the reactors were maintained at a similar level of around 2500 mg/L in MLSS by adjusting the strength of the feeding substrates. The reactors were operated under three simulated temperature variation strategies respectively in this study: 

I. Constant temperature process (20 ± 1 °C) in 1# reactor (R1).

II. Temperature rising period (from 20 °C to 35 °C by 1.5 °C per 3 days) and temperature recovery period (from 35 °C to 20 °C by 1.5 °C per 3 days) in 2# reactor (R2). 

III. Temperature dropping period (20 °C to 5 °C by 1.5 °C per 3 days) and temperature recovery period (5 °C to 20 °C by 1.5 °C per 3 days) in 3# reactor (R3).

### 2.3. Chemical Analysis

The concentration of COD, NH_4_^+^-N, TN, TP, mixed liquor suspended solids (MLSS) and mixed volatile liquor suspended solids (MLVSS) were measured according to Chinese NEPA standard methods (2002). Temperature, pH and DO were analyzed online with a WTW pH/ORP sensor pH3310 and a WTW DO sensor OXi340i (WTW, Munich, Germany).

### 2.4. EPS Extraction and Characterization

The tightly bound eps (tb-eps) and loosely bound eps (lb-eps) of the activated sludge were extracted by the modified heat extraction method described in details in a previous study [8]. Both the lb-eps and tb-eps extractions were analyzed for proteins (pns), polysaccharides (pss). The pns were analyzed by a uv/vis spectrophotometer following the modified bca method using bovine serum albumin (sigma) as the standard [9]. The pss were quantified by the phenol-sulfuric acid method [10]. The dna was quantified by nanodrop nd-1000 spectrophotometer (nanodrop technologies, Wilmington, United States).

### 2.5. DNA Extraction

Three different SBRs (i.e., R1, R2 and R3) were used in this study. The activated sludge underwent different simulated temperature variations in SBRs were sampled to extract the total microbial genomic DNA by using DNeasy^®^ PowerSoil^®^ Kit (QIAGEN, Chatsworth, CA, USA) according to manufacturer’s protocols. An exclusive name was assigned for each activated sludge sample. To be specific, the mixed activated sludge that was constantly operated R1 reactor at 20 °C at the initial stage of the experiment was named as A_1_. The activated sludge that were operated at 35 °C or 5 °C at the 33^rd^ cycle of simulated either increasing or decreasing of temperature in the R2, R3 reactors were assigned as A_2_ and A_3_, respectively. The activated sludge samples from R2 and R3 were named as A_5_, A_6_ respectively, when the water temperature of these SBRs was either decreased or increased respectively back to 20 °C at the 63rd cycle of simulated temperature variation. At the same time point, the activated sludge sample from the R1 reactor that was constantly operated R1 reactor at 20 °C was named as A_4_. The quantity and integrity and of the extracted DNA samples were confirmed by NanoDrop ND-1000 Spectrophotometer (NanoDrop Technologies, Wilmington, USA), Qubit^®^dsDNA Assay Kit in Qubit^®^2.0 Fluorometer (Life Technologies, CA, USA) and 1% agarose gel electrophoresis. The qualified RNA samples were stored at –80 °C before use.

### 2.6. Library Construction and High-Throughput Sequencing

A total amount of 1μg DNA per sample was used for the DNA sample preparations. The 350 bp paired-end sequencing libraries were generated using NEBNext^®^ UltraTM DNA Library Prep Kit for Illumina (New England Biolabs, Ipswich, MA, USA) following manufacturer’s instructions. Briefly, the DNA sample was fragmented by sonication to a size of 350 bp, then DNA fragments were end-polished, A-tailed, and ligated with the full-length adaptor for Illumina sequencing with further PCR amplification. PCR products were purified (AMPure XP system), quantified by Qubit^®^2.0 Fluorometer (Life Technologies, CA, USA) and diluted (2 ng/μL). Then, the insertion size of libraries was analyzed by Agilent 2100 Bioanalyzer and quantified using real-time PCR. Metagenomic sequencing was performed on the Illumina HiSeq platform (Novogene Bioinformatics Technology Co, Ltd., Beijing, China) with the sequencing strategy of 150 bp paired-end.

### 2.7. Quality Control and Sequence Assembly

In order to obtain the clean data for subsequent analysis, raw reads with >40 low-quality bases (quality threshold value ≤38) or with >10 ambiguous N bases were removed. In addition, the reads that shared an overlap of >15 bases with the adapter were also removed.

The clean data was assembled and analyzed with MEGAHIT software (version 1.0.4) to acquire scaffolds [11]. The assembled scaffolds were then interrupted from N connections to acquire scaftigs without N. The clean data from all samples were aligned to each scaftig, respectively, with Bowtie2 software (version 2.2.4) to acquire the reads not used, which were combined and then were processed as described above for mixed assembly [12]. Scaftigs less than 500 bp were filtered out.

### 2.8. Gene Prediction and Gene Catalogue Construction

The scaftigs (≥500 bp) were analyzed to predict the open reading frames (ORFs) using MetaGeneMark software (version 2.10) [13]. The predicted ORFs shorter than 100 nt were filtered out. CD-HIT software was adopted to remove redundancy and obtain the initial unique gene catalogues [14]. Sequences with ≥95% identity and 90% coverage were thought to be redundant and the longer one was retained as the representative sequence. The number of reads for ORFs aligned to each sample was obtained by aligning the clean data to initial unique gene catalogues with Bowtie2 software (version 2.2.4). Sequences that mapped to less than 2 reads were removed. The remaining gene catalogue (unigenes) was used for subsequently analysis. The absolute abundance and relative abundance of unigenes in each were calculated using the formula that showed in a previous study [15].

### 2.9. Taxonomic Assignment and Function Annotation

The species annotations of unigenes were obtained by blasting the non-redundant gene catalogue set with the MicroNR database [16]. All genes in the non-redundant gene catalogue set were aligned to the NR database using DIAMOND [17]. Taxonomic assignment of predicted genes was carried out using BLASTP alignment against the integrated NR database with an E-value cutoff of 10^−5^. The genes with the significant matches (defined by e-values ≤ 10 × e-value of the top hit) were retained to distinguish taxonomic groups. The Evolutionary Genealogy of Genes_Non-supervised Orthologous Groups (EggNOG) database and the Kyoto Encyclopedia of Genes and Genomes (KEGG) database were used to estimate the function and metabolic pathway of genes via the BLASTP program with an E-value cutoff of 10^−5^ [18]. The raw data of metagenome in this study were deposited in the NCBI with the BioProject ID of PRJNA591406, PRJNA591410, PRJNA591411, PRJNA591414, PRJNA591417, PRJNA591418.

## 3. Results and Discussion

### 3.1. Degradation Effect of Activated Sludge at Different Temperatures

**Figure 1:**
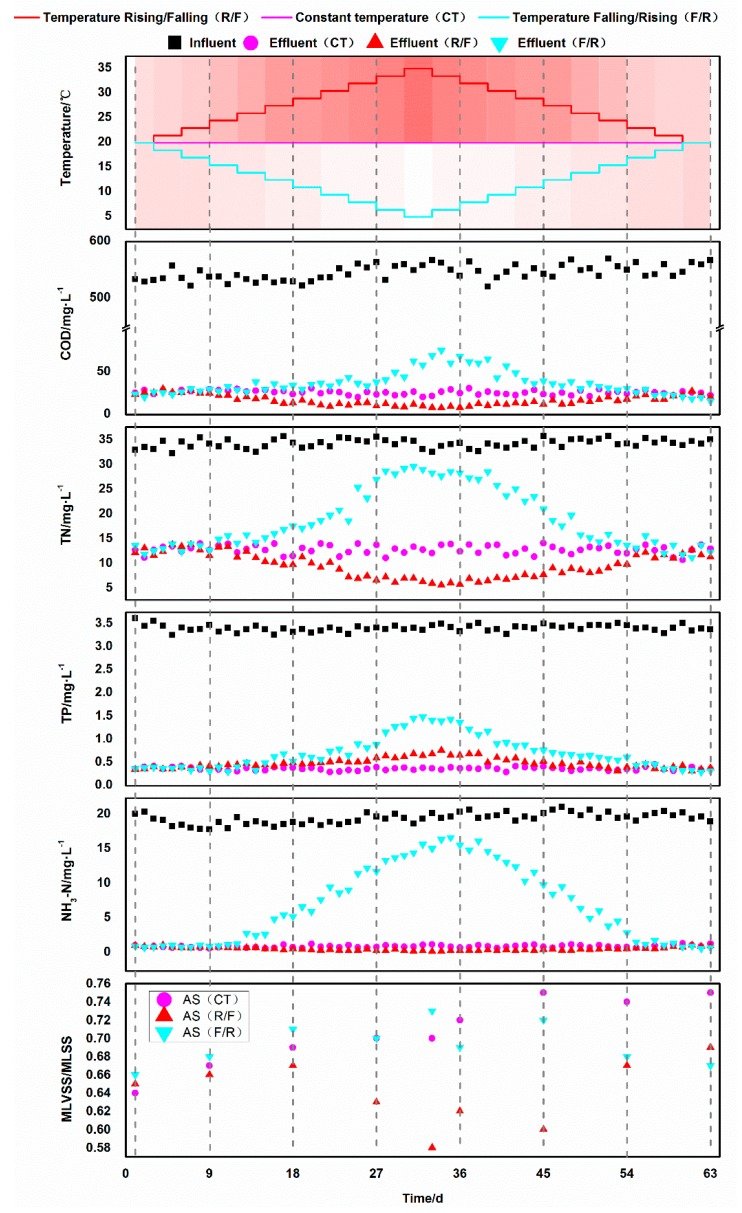
Removal effect of activated sludge system on pollutants under different temperature strategies.

After acclimatization for more than two months, the SBRs successfully accomplished the simulated temperature variation. The results for COD, TN, TP and NH_4_^+^-N removals are provided in Figure 1. It can be seen from Figure 1 that temperature variation in the SBR obviously impacted on the removals of COD, TN, TP and NH_3_-N of activated sludge.

#### 3.1.1. Chemical Oxygen Demand Removal

As shown in Figure 1, when the R1 reactor was operated at constant temperature (20 ± 1 °C), the COD of average effluent were remained at low level (around 26.0 mg/L) with the removal rate of around 95%. The effluent concentration of COD continued to decrease with temperature rose in the R2 reactor, and it decreased to 8.27–12.2 mg/L at 35 °C with the removal rate increased from around 95.5% to 97.8–98.5%. However, as the temperature dropped to 5 °C, the effluent COD concentration began to rise slowly, rising from 8.27–12.2 mg/L to 16.0–20.3 mg/L, and the average removal rate decreased by 2.6%. After 27 days of operation from 20 °C to 8 °C, high COD removal efficiency (>90%) was also maintained in in the R3 reactor. However, as the water temperature continued to decrease, the effluent COD concentration rose rapidly, resulting in a significant decrease in the removal rate. Especially when operation of the reactor lowered the water temperature to 5 °C on the 31st day, the effluent COD concentration rose rapidly to around 69 mg/L, and the removal rate was lower than 90%. According to Chevalier et al. [19,20], temperature has a significant effect on the adsorption performance, sedimentation performance, microbial growth and development, population composition and total oxygen transfer efficiency in the aerobic tank. The catalytic activity of the enzyme is inhibited by low temperature, thus the metabolic capacity of the microorganism in activated sludge is decreased. Therefore, the COD removal of the system is decreased.

#### 3.1.2. Nitrogen Removal

The effluent concentration of TN and NH_3_-N were evidently affected by the temperature (Figure 1). The ammonia water concentration of effluent was relatively stable and low in the R1 and R2 reactors during the whole experimental stage. The total nitrogen concentration in the R1 reactor was relatively stable in effluent (12.7 mg/L on average) and moderately high removal rate was achieved (62.3% on average). With the increase of water temperature from 32 to 35 °C started from the 25^th^ to the 39^th^ day, the TN concentration of R2 reactor effluent decreased from around 11.5–12.1 mg/L to 5.50–7.40 mg/L. Accordingly, high TN removal yield (>80.0%) was achieved in R2 reactor. The elevated water temperature increased the functional potentials of activated sludge. On the one hand, nitrification rate and ammonia nitrogen removal rate were improved. On the other hand, the denitrification nitrogen and phosphorus removal capacities of activated sludge were enhanced. The nitrate produced in the aerobic section of the water treatment system was consumed as an electron acceptor for denitrifying in the anoxic zone, thus the effluent NO_3_-N decreased and the TN removal rate increases [21]. As the temperature of the R2 reactor continued to decrease since the 34^th^ day, the TN effluent concentration steadily increased to the same level as the R1 reactor. In contrast, the declined water temperature inhibited the functional potentials of activated sludge. With the temperature continued to drop, the effluent of NH_3_-N concentration in the R3 reactor significantly increased to 15.2 mg/L on the 33^rd^ day, and the removal rate was significantly reduced (25.1%). The decrease of NH_3_-N removal also directly led to the effluent of TN concentration increased. The growth temperature for nitrifying bacteria is generally 4–45 °C. The optimum growth temperature of nitrite bacteria and nitric acid bacteria was is 35 °C and 35–42 °C, respectively. The optimum temperature for NOB (nitrite oxidizing bacteria) and DNB (denitrifying bacteria) were 30 °C [22]. As temperature declined, the metabolic activities of bacteria involved in nitrification and denitrification also decreased. Previous studies show that the nitrification rate was decreased significantly as temperature dropped [23,24]. With the continuous increase of temperature since the 36th day, the activity of microorganisms of the activated sludge in the R3 reactor gradually recovered. The removal ability of TN and NH_3_-N and were significantly increased to 65.1% and 95.5%, which was comparable with those of the activated sludge in the R1 reactor.

#### 3.1.3. Phosphate Removal

The removal rate of phosphate was modulated by either increased temperature or decreased temperature (Figure 1). The removal rate of phosphate was relatively stable and comparative in each reactor during the first three weeks despite of different simulated temperature variation strategies (Figure 1). Subsequently, the phosphate concentration of effluent in reactor R2 increased gradually to 0.689mg/L (removal rate of 80.1%) when temperature rose to 35 °C, and that of effluent in reactor 3 rose to 1.49mg/L (removal rate of 59.1%) in reactor R3 when temperature declined to 5 °C respectively. On the contrary, the total phosphorus concentration of effluent in the reactor R1 remained at a low level (0.396mg/L on average) and a high removal rate around 89.2% was maintained during the whole experimental period. The total phosphorus concentration of effluent in the reactor R2 and R3 decreased and became comparable with that in reactor R1 as the water temperature restored to 20 °C. The removal efficiency of phosphate in this study corroborated that reported by [25]. In general, temperature was a complex factor in the process of biological phosphorus removal of activated sludge mainly by the following three ways: (1) affecting the activity of phosphorus-accumulating bacteria; (2) affecting the microbial community structure in the activated sludge, such as the content of phosphorus-accumulating bacteria in the activated sludge during nitrification and acidification; (3) influencing possible chemical and physical processes, such as chemical precipitation process [26].

### 3.2. The Degradation Effect of the Activated Sludge under Typical Temperature in Single Cycle

**Figure 2:**
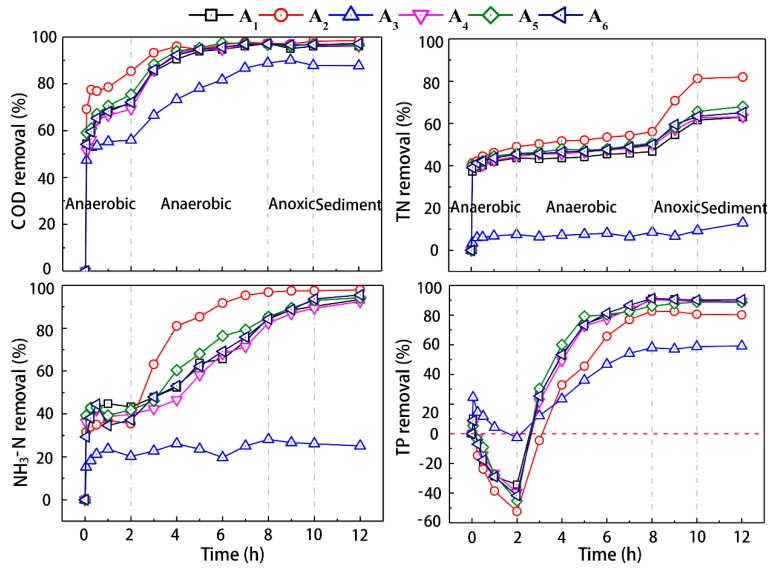
Removal of COD, TN, NH_3_-N and TP in activated sludge systems during typical cycles.

The degradation of COD for the activated sludge under different temperature in each typical cycle was shown in (Figure 2). In the anaerobic stage, the activated sludge system had a high removal rate for COD in wastewater in the first 30 min due to the dilution effect on the influent COD and the adsorption of the organic matters in simulated wastewater by activated sludge. The COD removal rate of activated sludge A_2_ (operated at 35 °C) at the end of anaerobic phase was much higher than those of other samples (Figure 2). Except for A_3_ (operated at 5 °C), the COD was almost removed in the wastewater in four hours with a high removal rate (above 90%). The removal of organic contaminants from wastewater by activated sludge mainly involved adsorption and microbial metabolism. With the temperature increases, the density, viscosity, and thickness of the interface solution film decrease, which makes the ion diffusion speed increase and accelerate the adsorption process. In addition, the microbial metabolic activity increased by a certain range of temperature in the microbial metabolic process. Therefore, temperature is an important parameter that greatly influence the COD removal in wastewater.

The removal of TN and NH_3_-N for the activated sludge under different temperature in each typical cycle was also shown in (Figure 2). The concentrations of TN and NH_3_-N decreased significantly due to the dilution of influent and the adsorption of flocs in the first 30 min in the anaerobic stage. Then, the ammonia content remained relatively stable (Figure 2), which was mainly ascribed to the inhibition of the nitrifiers by the extremely low dissolved oxygen (DO) in the anaerobic stage. The TN concentration decreased slowly, which was probably resulted from the anaerobic respiration of denitrifiers. The total nitrogen removal rate in the system was almost stable during the aerobiotic stage; while the ammonia content decreased gradually. Large amount of ammonia was likely converted into nitrate by nitrifiers and accumulated during aerobiotic stage, especially in activated sludge A_2_ system under increased significantly under 35 °C ascribing to the increased metabolic activity of nitrifiers (Figure 2) [27]. During the anoxic phase, the ammonia was continually removed with a rate comparable with that during the aerobiotic stage. The elevated TN removal rate in the anoxic was probably resulted from the denitrifiers converted the nitrate to organic nitrogen and nitrogen.

The total phosphorus concentration (TP) during the anaerobic phase decreased temporarily due to the dilution of influent. Then, the TP content in the water rose rapidly because of the released of phosphorus by phosphate-accumulating organisms (PAOs) (Figure 2). It was obvious that the rate of TP release was positively correlated with temperature. The significant decrease of TP during the aerobic stage was probably attributed to the absorption of phosphorus from sewage by PAOs. Comparable TP removal rates were achieved for these activated sludges operated under 20 °C (Figure 2). TP removal rates for these activated sludges operated under 20 °C were much higher than those obtained by the activated sludge under the temperature of 5 °C and 35 °C. This observation probably resulted from the inhibition of PAOs by either high temperature or low temperature as reported in a previous study [28].

### 3.3. Changes of EPS Composition and the Sludge Index of Activated Sludge under Different Temperature Variation

**Figure 3:**
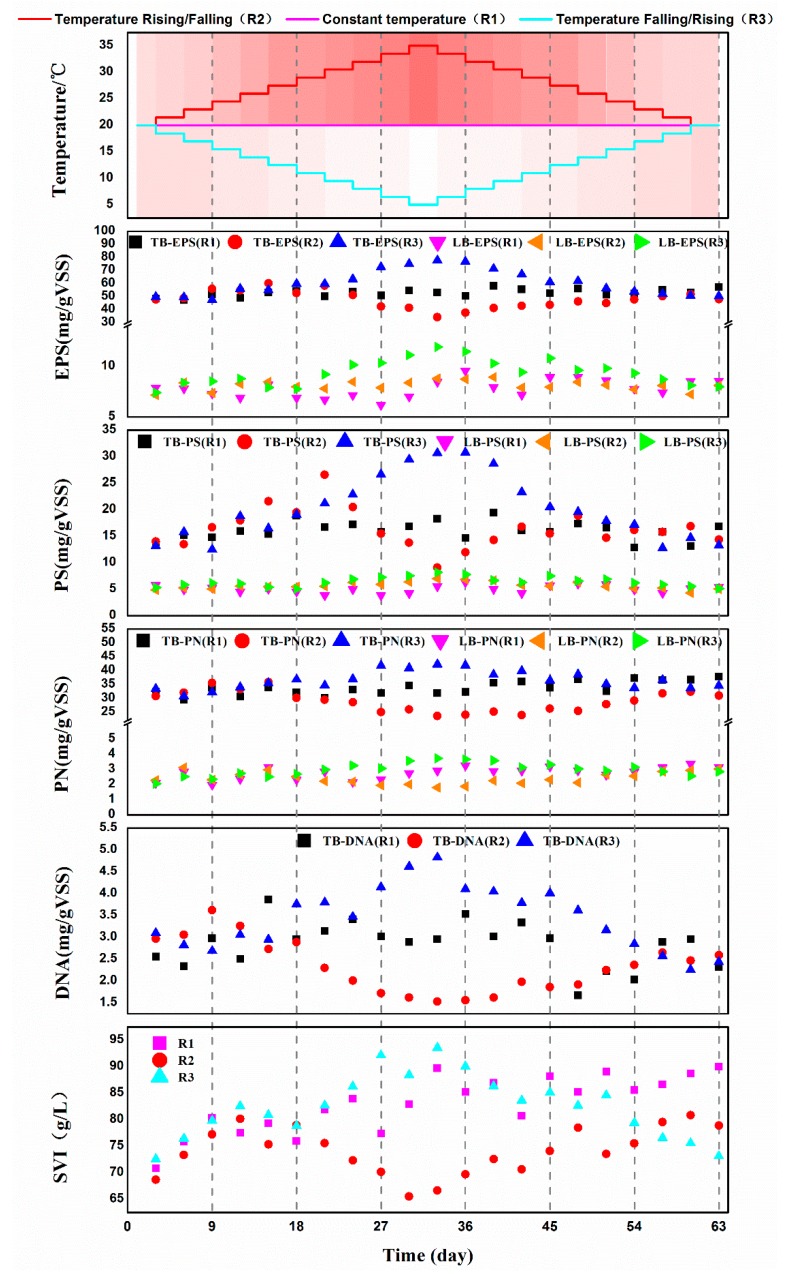
The variation of extracellular polymer substances (EPS) and sludge index of activated sludge in sequencing batch reactor (SBR) reactor under different temperature strategy.

Changes in the composition of extracellular polymer substances (EPS) and the sludge index of the activated sludge under different temperature were shown in (Figure 3). EPS is coated on the surface of microbial cells and filled in activated sludge to maintain the integrity of the activated sludge system. EPS has a significant impact on the physicochemical properties of activated sludge, including structure, surface charge, flocculation, sedimentation properties, dewatering performance and adsorption capacity [29]. Generally, the amount of tightly bound EPS (TB-EPS) was much higher than that of loosely bound EPS (LB-EPS) (Figure 3). The content of both TB-EPS and LB-EPS increased slightly by 17.6% and 8.35% to 57.12 mg/g VSS and 8.56 mg/g VSS respectively on the 63^rd^ day in the reactor R1 that was operated under 20 °C (Figure 3). The content of TB-EPS for activated sludge in the reactor R2 increased by 26.0% when the system temperature increased from 20 °C to 26 °C, then declined to 34.25 mg/g VSS when the temperature increased from 26 °C to 35 °C, and finally increased to 34.25 mg/g VSS when the temperature decreased to 20 °C. The content of both TB-EPS and LB-EPS increased by 56.20% and 60.80% to 77.80 mg/g VSS and 11.90 mg/g VSS, respectively in the reactor R3 that was operated under 5 °C as compared with that of under 20 °C (Figure 3). Different tendencies regarding on the change of tightly bound polysaccharide (TB-PS) were observed (Figure 3). No change was observed for the amount of PS for activated sludge in reactor R1. However, the amount of PS for activated sludge in reactor R2 increased steadily till the temperature rose to 30 °C, then started to decline and maintain a stable level. The amount of PS for activated sludge in reactor R3 increased to the maximum on the 33^rd^ day when the temperature declined to around 5 °C, then began to decrease gradually when the temperature returned to 20 °C. The tendencies regarding on the change of tightly bound protein (TB-PN) was very similar to that of the TB-EPS of each activated sludge sample (Figure 3). The amount of DNA was affected by temperature (Figure 3). The activated sludge at around 35 °C in reactor R2 had the minimum amount of DNA. However, activated sludge at around 5 °C in reactor R3 had the maximum amount of DNA. Generally, the sludge volume index (SVI) of activated sludge in reactor R1 increased before the 30^th^ day, then maintain almost stable till the end of the experiment (Figure 3). The increase of SVI was probably ascribed to the elevated EPS amount during the processing. The SVI of activated sludge in reactor R2 was increased before the temperature rose to 26 °C, then started to decline during the temperature continually rose to 35 °C, and thereafter increased with the temperature declined to 20 °C (Figure 3). The SVI of activated sludge in reactor R3 was increased as the temperature declined to 5 °C, then decreased to the level comparable with that of the original activated sludge sample A_1_.

### 3.4. Metagenomic Analysis

#### 3.4.1. Overview of Metagenomic Sequence

The effects of simulated temperature variation on the microbial community structure and functional genes of activated sludge were explored by metagenomic analysis. Around 6.11–6.78 Gbp clean data were obtained for each sample after removing adapter and low-quality reads, which generated 518, 038-619, 217 ORFs for each sample (Supplementary Table S1). Statistical results of sequencing data, assembly, and annotation (e.g., clean reads, N50, N90, contigs and ORFs) shows that the sequencing data and assembly efficiency obtained in this study are comparable to those of metagenomic studies on activated sludge [30,31]. Besides the larger number of shared genes (510,701) existed in all activated sludge samples, adequate number of specific genes (ranging from 9742 to 33,561) were also identified in each sample (Supplementary Figure S2). This implies that the microbial community structure and functional potentials of activated sludge were modulated by simulated temperature variation.

#### 3.4.2. Variation of Microbial Community Structure in Activated Sludge

A total of 147 phyla, 2,162 genera and 13,015 species were identified in those activated sludge samples, which indicates a high level of taxonomic diversity in the microbial communities of activated sludge samples. *Proteobacteria*, *Actinobacteria*, *Acidobacteria* and *Bacteroidetes* were the four dominant phyla for each activated sludge sample under simulated temperature variations. Comparable number of genera (2032–2062) or species (11,541–12,064) existed in each sample. The phyla with the relative abundance of the top 10 in each activated sludge sample were illustrated (Supplementary Figure S3). *Proteobacteria* is most dominant in each sample.

**Figure 4:**
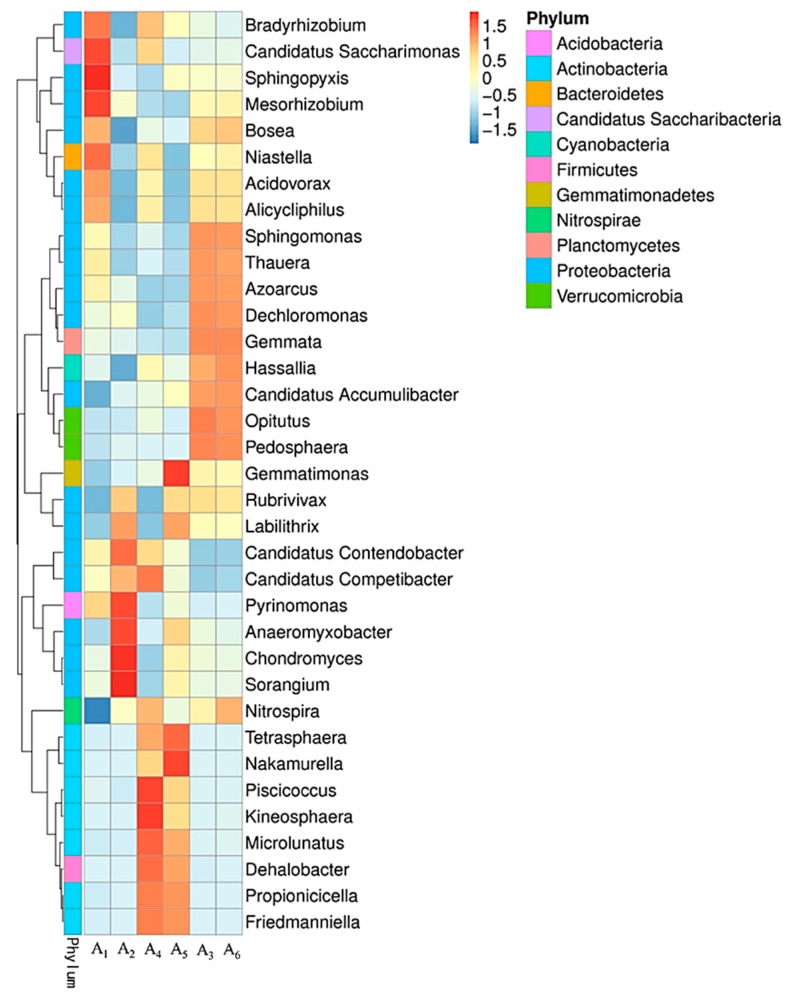
The clustering heatmap of the most abundant 35 genera in each activated sludge sample.

The predominant genera of activated sludge were significantly modulated by simulated temperature variation. Abundance of the top 35 genera in each of these activated sludge samples were selected to analyze their relative abundance (Figure 4). At the initial stage of the experiment, the dominant genera of activated sludge A_1_ were mainly consisted of *Sphingopyxis*, *Bradyrhizobium*, *Candidatus Saccharimonas*, *Mesorhizobium*, *Bosea*, *Niastella*, *Acidovorax*, *Alicycloiphilus* (Figure 4). In addition, the following genera of *Sphingomonas*, *Thauera*, *Azoarcus*, *Candidatus Contendobacter*, *Candidatus Competibacter* and *Pyrinomonas* were also existed with adequate proportion. *Sphingopyxis* and *Candidatus Competibacter* have the capacities to secrete EPS, which played important roles in accumulating phosphorus and stabilizing granule structure of activated sludge [32]. *Thauera*, *Acidovorax, Azoarcus* were involved in denitrification [33,34]. The abundant occurrence of these genera probably conferred the high TN, HN_3_-H, COD and total phosphorus removal activities of activated sludge originally operated under 20 °C (Figure 1). Some of these dominant genera observed in activated sludge sample A_1_ remained as the major genera in the activated sludge sample A_4_ that operated at constant temperature for 63 days (20 °C). Surprisingly, some new genera were shifted as dominant genera, such as *Piscicoccus*, *Kineosphaera*, *Microlunatus*, *Dehalobacter*, *Nitrospira*, *Tetrasphaera*, *Nakamurella*, *Propionicella* and *Friedmanniella* (Figure 4). *Piscicoccus* and *Kineosphaera* were involved in organic removal [35,36]. *Microlunatus* and *Tetrasphaera* played an important role in biological phosphorus removal [37,38]. *Nitrospira* was typical nitrite oxidation microorganisms [39]. Therefore, the TN, HN_3_-H, COD and total phosphorus removal activities were maintained despite the significant succession of microbial community.

Most of these dominant genera in original activated sludge sample A_1_ were shifted as minor genera in activated sludge sample A_2_ when the water temperature gradually increased to 35 °C except for the genera of *Candidatus Contendobacter*, *Candidatus Competibacter* and *Pyrinomonas* (Figure 4). Meanwhile, the following genera evolved as dominant genera, such as *Sorangium*, *Chondromyces*, *Anaeromyxobacter*, *Pyrinomonas*, *Labilithrix* and *Rubrivivax*. The functional roles for most of these dominant genera remains unknown. All these dominant genera existed in AS sample A_1_ together with some of the major genera in AS sample A_4_ were also observed as the dominant genera in the AS sample A_5_ in the reactor R2 when the water temperature declined gradually to 20 °C.

Although some of the predominant genera in AS sample A_1_ still existed as major components in the AS sample A_3_ in reactor R3 when water temperature declined to 5 °C, some genera evolved as dominant genera, such as *Dechloromonas*, *Gemmata*, *Hassallia*, *Candidatus Accumulibacter*, *Opitutus*, *Pedospaera* (Figure 4). *Opitutus* involved in nitrogen cycling [40]. *Candidatus Accumulibacter* and *Dechloromonas* involved in biological phosphorus removal [41,42]. It is interesting that the dominant genera in the AS sample A_6_ was almost identical with those existed in the AS sample A_3_ when the water temperature increased from 5 °C to 20 °C on the 63rd day. Therefore, the low TN, HN_3_-H, COD and total phosphorus removal activities of activated sludge under 5 °C were mainly attributed to the inhibitory effects on metabolic activities of microorganisms by low temperature other than the shifted microbial community structure.

### 3.5. Activated Sludge Microbial Gene Function Annotation

#### 3.5.1. COG Functional Classification and Functional Potential Analysis

Compared with previous studies [30,31,43], the functional gene abundance of the unknown classification was the highest, probably because more functional genes need to be explored. In addition to unknown functions, metabolically related functional genes have the highest abundance in each active sample compared to information storage and processing and cellular processes and signaling, which corroborates the previous studies.

**Figure 5:**
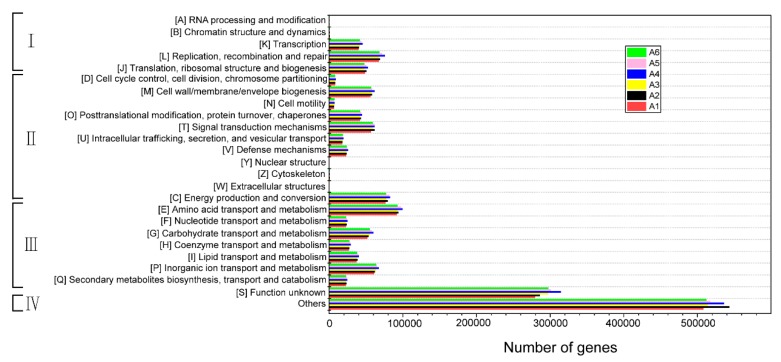
COG function classification of metagenome of activated sludge microbial communities.

The total abundance of functional genes in the activated sludge A_2_ was higher than those of the A_1_ and the A_3_ (Figure 5). It indicates that the microbial population increased in the activated sludge microbial community from the medium temperature (20 °C) to the high temperature (35 °C) stage, which led to the increase of functional gene abundance. It also verified that the unique gene abundance in A_2_ was the highest, and at the same time due to the higher functional gene abundance. The degree of A_2_ activated sludge shows a good treatment effect in the wastewater treatment process. When A_2_ and A_3_ activated sludge recovered to 20 °C stage, i.e., A_5_ and A_6_, the functional abundance of both were lower than the activated sludge A_4_ which kept in the constant temperature stage at the end of the experiment, which suggested overall similar functional potentials. At the same time, it can be seen through the detailed classification of the 24 functional groups that the functional gene abundance in the activated sludge samples at different stages showed extremely high similarity, indicating that within a certain temperature range the temperature had a limited effect on the metabolic activity of the microorganisms, which also confirmed in Figure 1. Even under low-temperature conditions, the activated sludge still had the ability of wastewater treatment [5].

#### 3.5.2. The Variation of Key Enzymes in Nitrogen Metabolic Pathways

Results showed that temperature had obvious impacts on the treatment of wastewater by activated sludge, especially for the removal of nitrogen pollution. However functional changing characteristics of associated enzyme genes had not been entirely explored in wastewater treatment systems under temperature variation. In the current study, 2,364 key enzyme genes were identified by high-throughput sequencing technology, which accounts for 23% of the total enzyme genes. And we investigated key enzyme genes involved in ammonification, nitrification, denitrification, nitrogen fixation and assimilatory nitrite reduction, including nitrilase, formamidase, carbamate kinase, glutamine synthetase, glutamate synthase, glutamate dehydrogenase, hydroxylamine reductase (*hcp*), ammonia monooxygenase (*amoA*, *amoB* and *amoC*), hydroxylamine oxidase (*hao*), nitrate reductase (*narB*, *narG* and *nxrA*), nitrite reductase (*nirK*, *nirS, nirA, nirB and nrfA*), nitric oxide reductase (*norB* and *norC*), nitrous oxide reductase (*nosZ*), assimilatory nitrite reductase, and nitrogenase (*nifD*, *nifK*, *nifH* and *anfG*).

**Figure 6:**
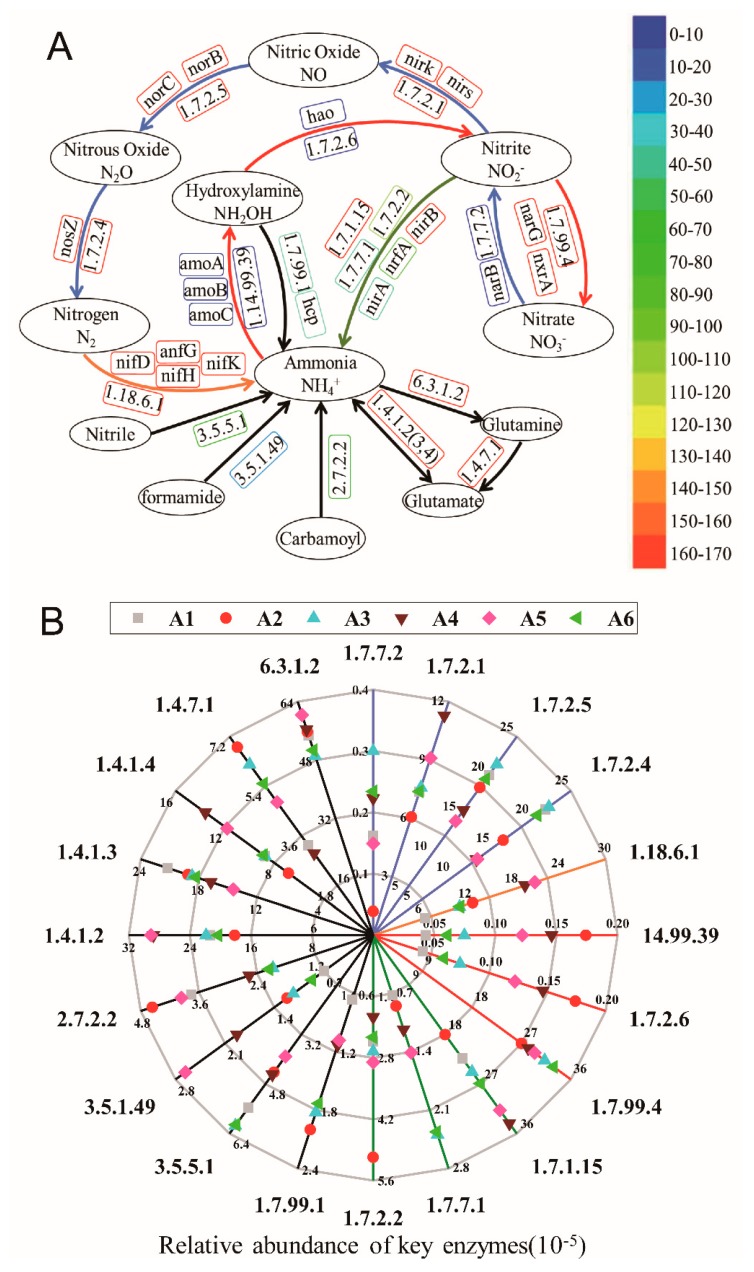
Analysis the key enzymes involved in nitrogen metabolic pathways in activated sludge (**A**) Nitrogen metabolic pathways including the processes of ammonification (black), nitrification (red), denitrification (blue), nitrogen fixation (orange), and assimilatory nitrite reduction (green).; (**B**) Relative abundances of key enzymes.

The enzyme genes related to ammonification had the highest abundance despite of changing temperature, especially for gene encoding glutamine synthetase (EC:6.3.1.2) which involved in a dominate N-immobilization pathway of microorganism (Figure 6A) [44,45,46]. With the temperature raising from 20 °C to 35 °C, the abundance of *amoCAB* genes encoding ammonia monooxygenase (EC:1.14.99.39) increased by 305.8%, which indicated that the increase of temperature may be beneficial to the activity of ammonia monooxygenase. The result is in agreement with the low levels of effluent NH_4_^+^-N observed throughout the experimental period (Figure 1). Since NH_4_^+^-N in water was positively charged and the inside of the cell membrane is negatively charged, NH_4_^+^-N was more likely to be actively transported into the cell than NO_3_-N, and the amino acid was directly synthesized without reduction. This indicates that the NH_4_^+^ assimilation capacity of the activated sludge was high under different temperatures, especially under higher temperatures (20 °C-35 °C). Meanwhile, all enzyme genes associate with denitrification were reduced (Figure 6B), which might be resulted from lack of carbon source. Carbon source as electron donor would promote denitrification [47], with the increasing of temperature the system might be short of carbon source due to the rapid growth of microorganisms. Thereby, microorganisms can only consume EPS as carbon source [47,48], which is consistent with the decline of the EPS at around 35 °C. As the temperature dropped from 20 °C to 5 °C, the abundance of enzyme genes related to nitrogen metabolism was raised except for carbamate kinase (EC:2.7.2.2), glutamate dehydrogenase (EC:1.4.1.3), glutamine synthetase (EC:6.3.1.2). The abundance of nitrite reductase (*nrfA*) which is involved in the reduction of nitrite to ammonia raised by 230.6%, and hydroxylamine reductase (*hcp*) related to the reduction of hydroxylamine to ammonia raised by 174.9%, which might lead to a decline in the NH_4_^+^-N removal rate. Since the expression of functional genes of either single strain or microbial consortium were altered by various physicochemical parameters [49,50,51,52]. Therefore, it is necessary to compare the profiles of expressed functional genes under different simulated temperature variation by metatranscriptomic analysis in near future.

## 4. Conclusions

The effects of simulated seasonal temperature variation on the activities of activated sludge were studied in laboratory-scale reactors with simple and controllable experimental conditions in this study. Data shows that higher temperatures (20 °C to 35 °C) consolidate the TN and COD removal activities of activated sludge and inhibit the TP removal activity. On the contrary, lower temperatures (below 20 °C) inhibit the TN, HN_3_-H, TP and COD removal activities of activated sludge. Both the EPS composition (e.g., total amount, PS, PN and DNA) and sludge index of activated sludge were altered by simulated seasonal temperature variation. Metagenomic analysis shows that the simulated seasonal temperature also modulated the microbial community structures, which should partially interpret the observations regarding on the changes of activities and EPS composition of activated sludge. Some genetic characteristics of the microbial community, e.g., nitrogen metabolic pathways, are possibly influenced by simulated seasonal temperature variation. Collectively, these results add insights into the microbial community structures and the metabolic processes associated with the activities of activated sludge under simulated seasonal temperature variation.

## Supplementary Materials

The following are available online at https://www.mdpi.com/2076-2607/7/12/713/s1, Table S1: Statistics of the sequencing data, assembly and annotation; Figure S1 Schematic of lab-scale device (A) and operation cycle of SBR reactor (B). Figure S2 Venn diagram analysis of the gene numbers identified in each sample. Figure S3 The top 10 abundant phyla in each activated sludge sample.
