# Supplementary material for: Metagenomic Insights into the Effects of Seasonal Temperature Variation on the Activities of Activated Sludge
Source: Microorganisms. 2019 Dec 17;7(12):713. doi: 10.3390/microorganisms7120713 (PMC6956059; doi:10.3390/microorganisms7120713)

Supplementary table S1. *Statistics of the sequencing data, assembly and annotation.*

Sample	Raw Data (Mb)	Clean Data (Mb)	Scaftigs	N50 (bp)[a]	N90 (bp)[b]	ORFs	Effective (%)
A1	6,755.32	6,712.61	315,681	1,194	563	537,778	99.368
A2	6,161.06	6,114.40	374,853	1,078	558	619,217	99.243
A3	6,797.13	6,775.69	337,058	1,214	572	553,884	99.685
A4	6,752.60	6,726.10	347,902	1,043	558	560,427	99.608
A5	6,293.87	6,284.21	331,720	1,100	559	540,384	99.847
A6	6,518.82	6,500.91	315,896	1,190	571	518,038	99.725

[a,b]N50(N90), the length of the smallest scaftigs in the set of largest scaftigs that have a combined length that represents at least 50%(90%) of the assembly.

Supplementary Fig. S1 Schematic of lab-scale device (A) and operation cycle of SBR reactor (B).

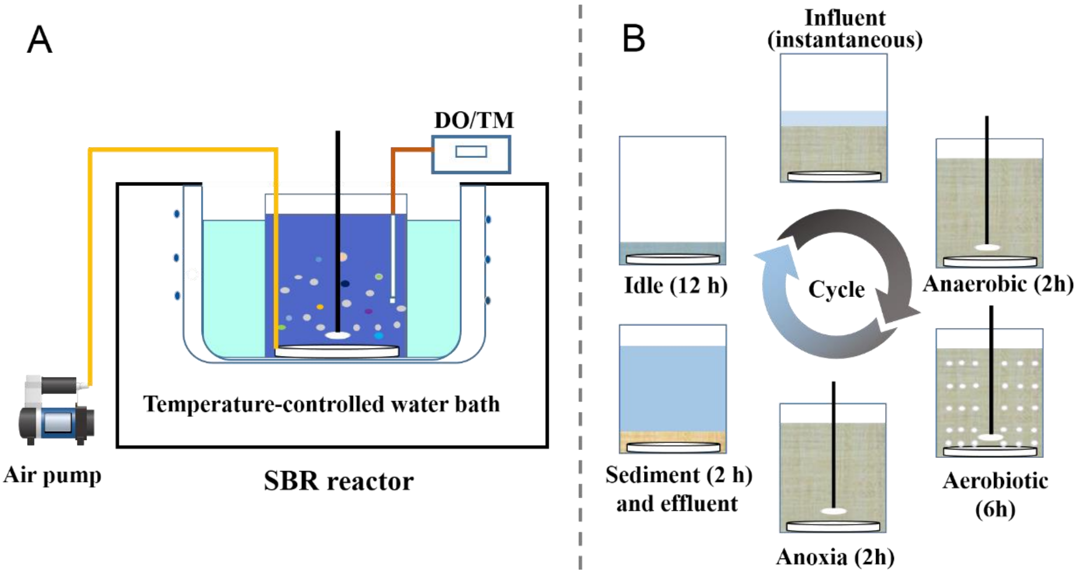

Supplementary Fig. S2 Venn diagram analysis of the gene numbers identified in each sample.

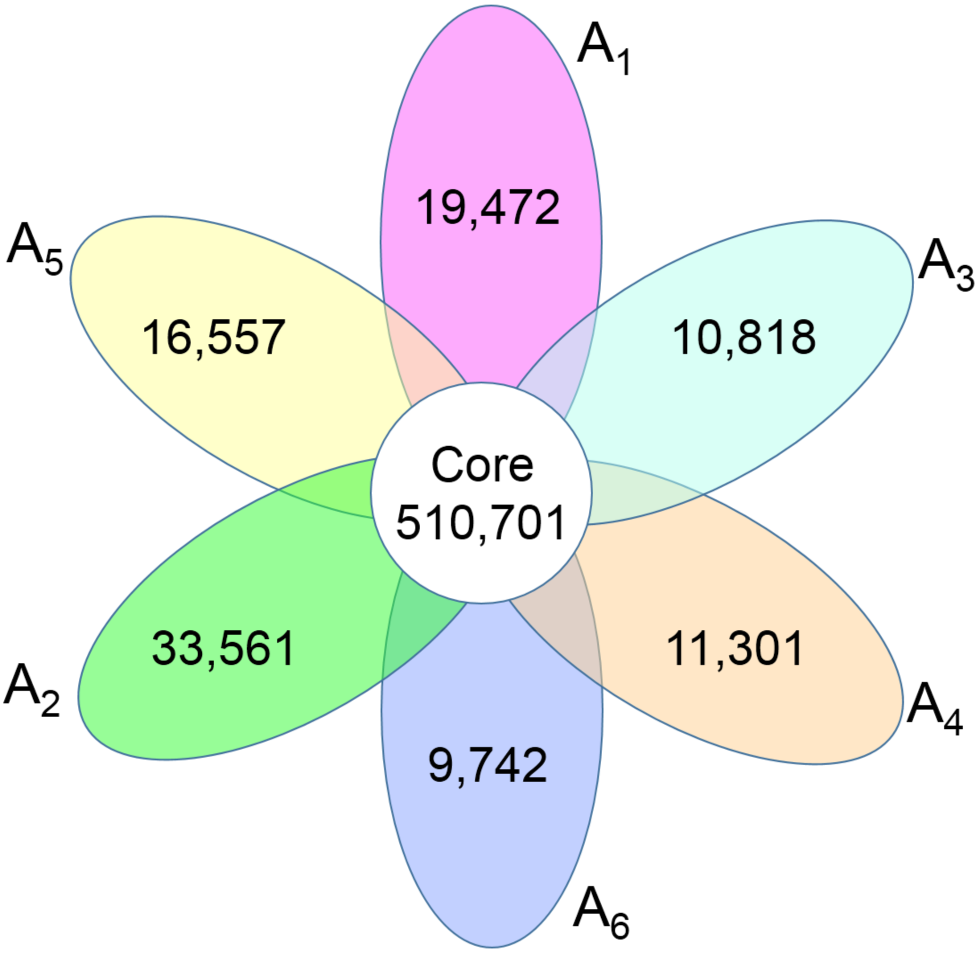

Supplementary Fig. S3 The top 10 abundant phyla in each activated sludge sample.

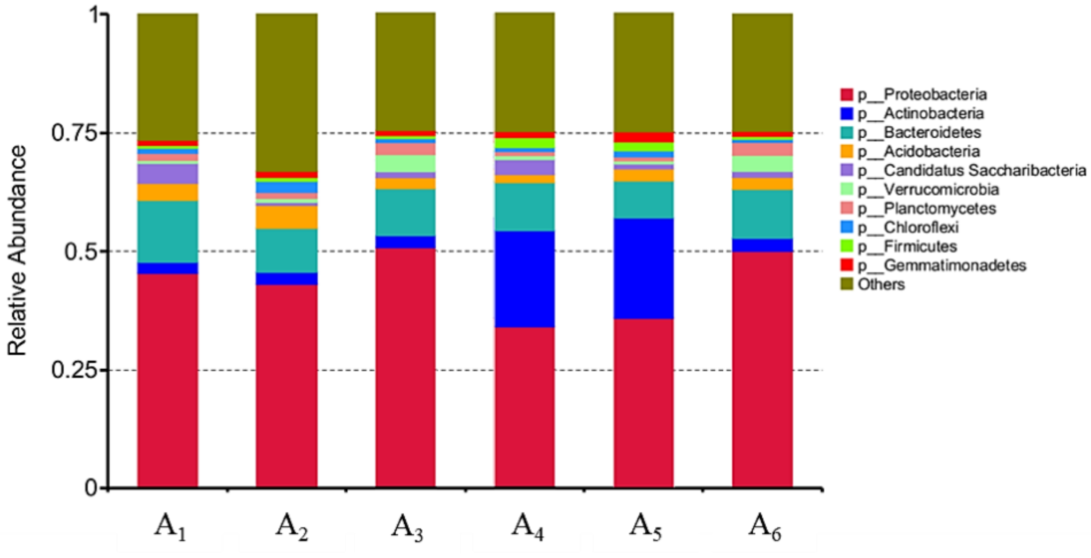

Supplement: Supplementary file 1 [file microorganisms-07-00713-s001.pdf]
